# Potential of polygenic risk scores for improving population estimates of women’s breast cancer genetic risks

**DOI:** 10.1038/s41436-021-01258-y

**Published:** 2021-07-06

**Authors:** Michael Wolfson, Steve Gribble, Nora Pashayan, Douglas F. Easton, Antonis C. Antoniou, Andrew Lee, Sasha van Katwyk, Jacques Simard

**Affiliations:** 1 School of Epidemiology and Public Health, University of Ottawa, Ottawa, Canada; 2 Department of Applied Health Research, University College London, London, UK; 3 Department of Public Health and Primary Care, Cambridge, UK; 4 Department of Oncology, University of Cambridge, Cambridge, UK; 5 Department of Molecular Medicine, Université Laval, Quebec City, Canada; 6 CHU de Quebec—Université Laval Research Center, Quebec City, Canada

## Abstract

**Purpose:**

Breast cancer risk has conventionally been assessed using family history (FH) and rare high/moderate penetrance pathogenic variants (PVs), notably in *BRCA1/2*, and more recently *PALB2*, *CHEK2*, and *ATM*. In addition to these PVs, it is now possible to use increasingly predictive polygenic risk scores (PRS) as well. The comparative population-level predictive capability of these three different indicators of genetic risk for risk stratification is, however, unknown.

**Methods:**

The Canadian heritable breast cancer risk distribution was estimated using a novel genetic mixing model (GMM). A realistically representative sample of women was synthesized based on empirically observed demographic patterns for appropriately correlated family history, inheritance of rare PVs, PRS, and residual risk from an unknown polygenotype. Risk assessment was simulated using the BOADICEA risk algorithm for 10-year absolute breast cancer incidence, and compared to heritable risks as if the overall polygene, including its measured PRS component, and PV risks were fully known.

**Results:**

Generally, the PRS was most predictive for identifying women at high risk, while family history was the weakest. Only the PRS identified any women at low risk of breast cancer.

**Conclusion:**

PRS information would be the most important advance in enabling effective risk stratification for population-wide breast cancer screening.

## INTRODUCTION

Risks of breast cancer (BC), are known to be influenced by genetic susceptibility, with three general sources of information for assessing this susceptibility: (1) family history (FH); (2) high-risk but rather uncommon deleterious variants in several susceptibility genes, such as *BRCA1* and *BRCA2* (pathogenic variants, PVs); and most recently (3) common susceptibility variants, which can be efficiently combined into a polygenic risk score (PRS). Mavaddat et al. [1] recently developed a 313 single-nucleotide polymorphism (SNP)-based PRS, accounting for approximately 20% of the polygenic risk of BC.

In this context, there is considerable interest in applying as much of this genetic information as possible for stratified BC screening and prevention strategies [2], including possible changes to breast cancer screening programs. BC screening in most developed countries is typically offered to women based on their age, usually starting at age 50. Assessing a women’s genetic risk would enable organized screening programs to offer BC screening starting at earlier ages to high-risk women, and possibly later ages and/or lower frequencies to low-risk women [3].

Considerable debate remains whether the net benefits of such changes to BC screening programs would be worthwhile in terms of earlier BC detection, false positives, overdiagnosis, costs, feasibility, and acceptability, though recent studies do suggest that tailoring BC screening programs to individual risks would likely be cost-effective compared to current “one size fits all” age-only based screening programs [3, 4]. Central to such a cost-effectiveness assessment is (1) the comparative value of FH, PV, and PRS for risk prediction, which in turn requires (2) valid estimates of the joint distribution of these risk factors in the population. It is these latter two questions that are the focus of this paper.

Population-wide estimates of the joint distribution of women’s FH, PV, and PRS are unavailable, so recent cost-effectiveness evaluations [3–6] have had to rely on partial and more approximate approaches. From a health provider or public policy perspective, though, the question is how such risk-based screening would work on a population level, taking account of the full range of variations in women’s risks and the various ways of capturing this risk.

In this analysis, we first provide a realistic estimate of the population joint distribution of FH, PV, and PRS for Canada. To do this, we make use of BOADICEA [7], a well validated risk model that incorporates FH, PV, and PRS and is widely used to predict a woman’s BC risk [8]. We then examine which genetic information from amongst FH, PV, and PRS would be most important for assessing a woman’s BC risk at the level of detail usable by BOADICEA, including the possibility of very detailed FH information for all first- and second-degree relatives, not only for BC, but also for prostate, ovarian, and pancreatic cancer, as these are informative for BC risk and can be taken into account by BOADICEA [9].

## MATERIALS AND METHODS

These results were derived from our novel genetic mixing model (GMM), which produces realistic population estimates of the full multivariate joint population distribution of FH, PV, and PRS, including correlations, since there are no adequately representative directly observed unbiased population surveys of sufficient sample size. GMM embodies the core BOADICEA risk algorithms by incorporating its relevant software code.

### Development of GMM

GMM is an interacting agent, continuous time, Monte Carlo microsimulation model. It simulates key demographic events—union formation and dissolution, nulliparity and parity-specific fertility—as well as relationships that reflect blended families including half-siblings, and all-cause mortality. This builds on the software architectures and sociodemographic dynamics parameters of Statistics Canada’s Lifepaths [10] and the Canadian Partnership Against Cancer’s HPV component of the Cervical Cancer [11] models. Each simulation starts with 4 million men and women whose ages are distributed according to the recent Canadian population age structure. Each starting individual is endowed with a randomly assigned genotype (PRS + presence or absence of PVs in each of the five genes + residual *unobserved* polygenic risk) using the population prevalences in BOADICEA. For the purpose of these analyses, the population prevalences of the PVs in Canada were assumed to be those in the standard implementation of BOADICEA.

As (continuous) time unfolds in a simulation, men and women form unions, have children and stepchildren, pass on their genetic endowments, possibly have incident cancers, and eventually die. Each simulation continues for about 200 years, so at the end there is a representative cross-sectional sample of women where all their first- and second-degree relatives have also been simulated at least up to the age of the proband’s risk assessment, and where each family member in the proband’s pedigree has a biologically appropriate genotype.

GMM retains pointers to each relative (parent, grandparent, child, aunt, uncle, etc.), tracking the evolution of each woman’s pedigree through time, enabling FH to be extracted at any specific age for postulated BC risk assessment. By construction, the simulated distribution of pedigrees reasonably reflects correlations of both demography and genetics.

Union formation and dissolution and fertility dynamics are based on multivariate transition probability density functions as in the Statistics Canada LifePaths [10] and Canadian Partnership Against Cancer HPV models [11]. Couples in first and/or subsequent stable unions generate births based on observed fertility rates conditional on a woman’s single year of age and parity at each moment during her lifetime, enabling realistic distributions of age differences between mothers and their children, numbers of female siblings, and age differences between women and their siblings.

For genetic susceptibility, GMM is based on the most recent version of BOADICEA [7], including both variants in *BRCA1*, *BRCA2*, *PALB2*, *CHEK2*, and *ATM* (PV), and a polygenic component that models the effects of a large number of variants of small effect. GMM, following the methodology described in [7], divides the overall polygenotype into two independent normally distributed components: “known," which is measured by the PRS and assumed to account for 20% of polygenic risk [1], leaving 80% as “unknown," which represents the residual familial aggregation of BC risk not attributable to PVs or to the PRS as specified.

Individuals’ PVs are initially assigned to the founding population based on the assumed allele frequencies [9] and then transmitted to offspring according to Mendel’s first law. Two polygenotypes are assigned to each individual (male and female) in the starting population by drawing randomly one number from a Gaussian distribution with variance γσ^2^ for the known component (i.e., the PRS), and a second number from an independent Gaussian distribution with variance (1 – γ)σ^2^ for the unknown residual component, where γ = 0.2, i.e., the PRS is taken to represent 20% of the variability in the overall polygenotype. Subsequently, the two polygenotypes for each newborn child are drawn from each of these two Gaussian distributions, one for the PRS and the other for the unknown residual portion of the polygenotype, with means for each of these two distributions set equal to averages of the values for that child’s parents, with variances γσ^2^/2 and (1 – γ)σ^2^/2, respectively. These formulae ensure that the polygenic distributions are stable over generations. GMM then simulates BC incidence for every individual in the population, thus generating the pedigrees to enable FH to be constructed for any given proband.

It should also be noted that GMM explicitly simulates the genetic risks and incidences of ovarian, prostate, and pancreatic cancers. It uses exactly the same age-specific incidence and relative risks as the internal BOADICEA algorithms. The resulting simulated cancer occurrences are then made available to BOADICEA by the GMM algorithms to the extent that the risk assessment scenario being simulated (e.g., no FH, or FH only for first-degree relatives) is posited as being provided by the proband as part of her FH.

### Application

**Fig. 1:**
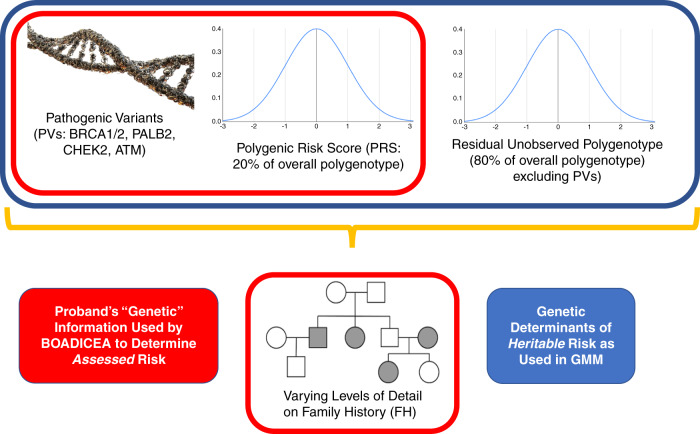
Kinds of information used by the genetic mixing model (GMM) to compute *assessed* and *heritable* risks of breast cancer incidence. Items outlined in red were used as inputs to BOADICEA for computing the *assessed* risk; those outlined in blue were used by other BOADICEA algorithms for computing the *heritable* risk.

GMM performs two parallel calculations of BC risk for each woman at the age posited in a given simulation scenario for her risk assessment. First, the *assessed* risk is calculated using BOADICEA for the combination of the FH, PV, and PRS information provided to BOADICEA. Note that this information may be incomplete. (See the following section for a description of parameter scenarios.) Second, the *heritable* risks are calculated using the same internal BOADICEA risk algorithm. These heritable risks are those for each woman associated with the five PVs, the PRS, plus the “unknown” residual portion of her polygene. For these latter calculations, FH is irrelevant, since cancer risk is assumed independent of that observed in relatives, once the proband’s genotype, PV and PRS and residual polygenotype, are fully known. The differences in the information used to compute assessed and heritable risk are illustrated in Fig. 1. Note that the *heritable* risk measure is not the “true” or “overall” risk. It is the risk if all inherited risk factors were known precisely at birth; it does not consider other known risk factors, for example parity or breast density (specifically their nonheritable components), nor other unknown risk factors.

## RESULTS

### Genetic risk assessment scenarios

From a population perspective, the two central program or policy parameters of a risk-based stratified screening program are (1) the age at which women’s risks are assessed, and (2) the threshold defining “high risk.” A further parameter is the risk threshold for being classified as “low risk.” For ages at risk assessment, we focus on ages 30 to 50 in five-year steps. Since average absolute cancer incidence risk increases nonlinearly with age, and we are going to be evaluating risk stratification occurring at different ages, it is important for the risk thresholds to be age varying. Therefore, we have expressed these thresholds as multiples of the age-specific absolute 10-year risk. For the high-risk thresholds, we consider a series of multiples: 2.0, 2.5, 3.0, 3.5, and 5.0, while for the low-risk threshold we consider similarly defined risk thresholds of 0.5 and 0.8 times the age-specific absolute 10-year risk.

Given a set of these parameters, as well as the specific set of (possibly partial) genetic information provided by each woman to BOADICEA for her individual-level risk assessment, GMM produces the population distribution of predicted *assessed* genetic risks. BOADICEA by design, and hence GMM in its use of the BOADICEA algorithm, also produces appropriate unbiased risk assessments even when passed only partial information on the proband.

For FH, we explored four possible scenarios: (1) no FH information provided at all by the woman (“none”), (2) a simple yes/no for whether the proband’s mother was diagnosed with BC at age ≤45 (FHm45), (3) full information for first-degree relatives only (FH1), or (4) full information for both first- and second-degree relatives (FH2).

**Fig. 2:**
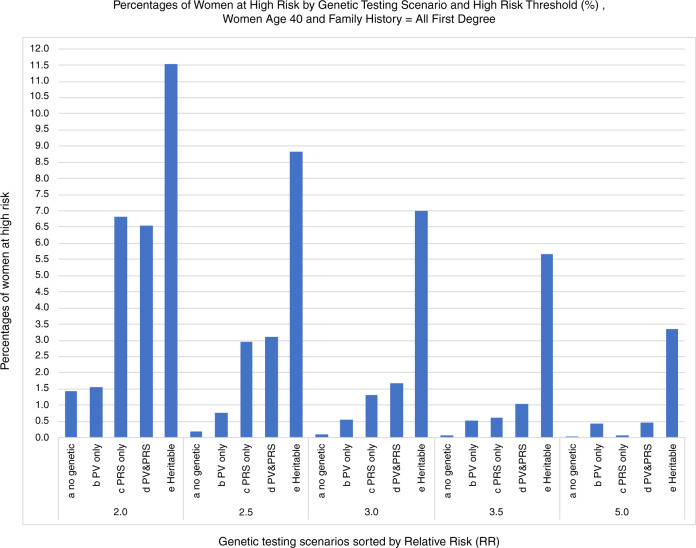
Percentages of women at high risk by Genetic Testing Scenario and High Risk Threshold. All women are assessed at age 40 and family history information provided to BOADICEA is always first degree relatives only. Results are sorted along the horizontal axis with inner grouping by genetic testing scenario (a to d) plus the “heritable” risk (e), and then sorted in the outer grouping by high-risk threshold (multiple of ten-year absolute risk at age 40). “Heritable” denotes women classified as high risk assuming all of their pathogenic variant (PV), polygenic risk score (PRS), and the unobserved residual portion of the polygenotype were known.

Figure 2 shows the percentages of women identified as high risk depending on which directly measured genetic information is provided to BOADICEA for risk assessment, assuming the assessments are all at age 40 and include full family history information for all first-degree relatives (FH1). At the outermost level, a series of five high-risk thresholds ranging from 2.0 to 5.0 times age 40 absolute risk of BC incidence is displayed along the horizontal axis. Then within each high-risk threshold category, the *assessed* risk for four combinations of genetic information ([PV yes/no] × [PRS yes/no]) plus as a fifth, the estimated full *heritable* risk, are shown.

At all high-risk thresholds (Hi-RT), the proportion of women identified as high risk based on *heritable* risk is substantially larger than any of the proportions based on assessed risks. As expected, for each kind of BOADICEA input, the percentages of women *assessed* as high-risk decline as the high-risk threshold increases. With a high-risk threshold of 5.0, less than half of one percent of women would be *assessed* as high risk (bars a to d in the rightmost set of vertical bars); this amounts to less than one-sixth of all women who are *heritably* at high risk (bar e in this same rightmost set of bars).

For all the high-risk thresholds, the percentages *assessed* as high risk is smallest when neither PRS nor PV is taken into account. If PVs are included (but not PRS), the percentage of high-risk women increases only slightly at the 2.0 high-risk threshold (Hi-RT), but more markedly as Hi-RT increases. In contrast, providing PRS but not PVs to BOADICEA increases the percentages of women assessed as high risk at all thresholds. The incremental importance of PRS alone compared to PV information alone declines, and eventually reverses as the risk threshold increases to the last ≥5.0 relative risk threshold.

**Fig. 3:**
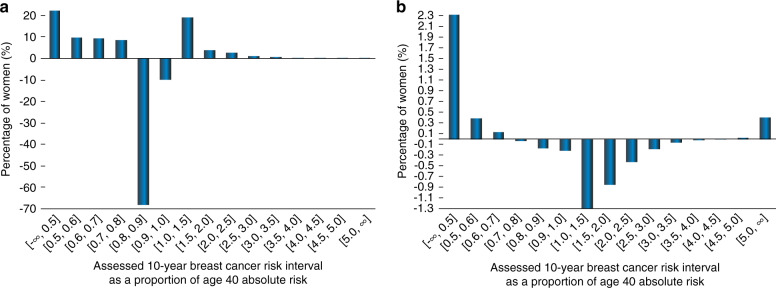
Net reclassification of the assessed risk at age 40 for two scenarios for adding the genetic information to compute the assessed risk for breast cancer. In both scenarios, first-degree family history (FH1) is taken as known. **a** Results obtained by including the polygenic risk score (PRS) given the pathogenic variant (PV) is already used for the risk assessment. **b** Results obtained by including the PV information given PRS is already used. Note the very different vertical axis scales.

Perhaps counterintuitively, if the PRS is known, then including PVs actually *reduces* the percentage assessed as high risk when Hi-RT is lowest at 2.0, but *increases* the percentage of women assessed as Hi-RT for higher risk thresholds, comparing the heights of the c and d vertical bars when scanning across the groups of bars from lower to higher Hi-RTs. This apparent paradox is explained in connection with Fig. 3.

Figures 3a, b show the extent to which women’s risks are reclassified under two scenarios for the information they would provide to BOADICEA, assuming assessment at age 40 and full FH1 information has been provided, with Fig. 3a showing the incremental effects on assessed BC risks of providing PRS information given PV has already been provided, and Fig. 3b providing PV given PRS has already been provided. The proportion of women in the highest risk interval (≥5.0 times age 40 absolute risk) increases with the addition of PV information as indicated by the rightmost vertical bar in Fig. 3b, an increase in the proportion of women classified as high risk of about 0.5%. Moving to the left we see for example in the [2.5, 3.0] risk interval that there is a decrease in the percentage of women of about 0.2% while in the [2.0, 2.5] risk interval, the decrease is over 0.4%. As a result, the sum of the decreases in the percentages of women in the risk intervals from 2.0 to <5.0 is greater than the increase in the percentage for the top open-ended ≥5.0 risk interval. Thus, for a risk threshold = 2.0 times age-specific absolute risk, the net effect of adding PV information to PRS information for purposes of risk assessment is negative; the addition of PV information in this case more often moves women down rather than up in terms of their assessed risk.

(Note, though, that these are *net* amounts of movements or reclassifications across risk intervals; for any one risk interval, some women will have moved in while others will have moved out. While it would be interesting to visualize the gross reclassification flows, this is not practically feasible. Instead of simply comparing two separate Monte Carlo microsimulation runs of GMM, as has been done here, it would be necessary to enlarge GMM significantly in such a way that each woman in a simulation could at any given age have multiple calls to BOADICEA to assess her risk under varying scenarios for the information provided to BOADICEA. This is beyond the intended scope of GMM.)

In contrast to Fig. 3b, Fig. 3a shows the net effects when PRS is added to the information provided to BOADICEA when PV has already been provided. These effects are an order of magnitude larger than in Fig. 3b, with almost a tenfold larger scale on the vertical axis. Almost all women move from having assessed risks just below average—some to higher risk intervals, but more often to lower risk intervals. Virtually no women move to the highest risk intervals (≥3.5) with the addition of the PRS information.

The next three figures all show the univariate distributions of both assessed (thick bars) and heritable risks (thin bars) in horizontal stacked bar chart form. These thick and thin bars are further color-coded to show either the two low-risk intervals (dark and light blue for very low and low respectively) or the two high-risk intervals (red and orange for very high and high respectively). In between are those residually at “average” risk. Vertically, each figure shows a series of scenarios with different combinations of age at risk assessment, the kind of FH information provided to BOADICEA, whether or not PV and/or PRS information is provided to BOADICEA, and the high-risk threshold expressed relative to the age-specific absolute BC risk.

**Fig. 4:**
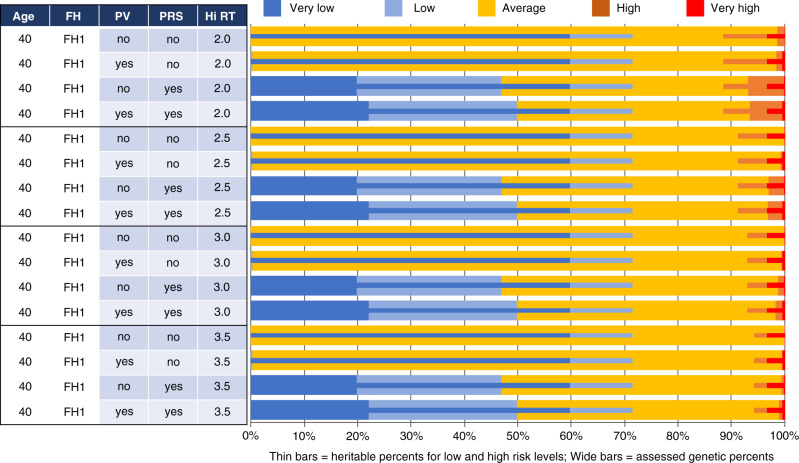
Univariate assessed and heritable breast cancer risk distributions by genetic information provided to BOADICEA and by high-risk threshold (Hi-RT, the multiple of the age-specific absolute risk of breast cancer incidence). In all cases, the risk assessment is for women aged 40 with their full first-degree family (FH1) proband information provided to BOADICEA. The rows are sorted by Hi-RT, and within the Hi-RT groups by whether the polygenic risk score (PRS) and/or pathogenic variant (PV) information has been used.

The four sets of rows in Fig. 4 correspond to various high (age-specific) relative risk thresholds (Hi-RT), and within each Hi-RT all four combinations of (PRS yes/no) × (PV yes/no). Without any PRS information provided to BOADICEA, no one is assessed (thick bars) at either “very low” (<0.5) or “low” (0.5 to 0.8) risk. With PRS (but not PV) provided, approximately 20% of women are assessed as very low risk, and almost 50% as low risk. These proportions all increase by about three percentage points when PV is also included in the BOADICEA assessment. First-degree family history (FH1), in the absence of any genetic testing (i.e., no PV or PRS), only identifies a very small fraction of women as high risk, and then only when the high-risk threshold is 2.0. For high-risk thresholds at 2.5, 3.0, or 3.5 times age 40 absolute BC risk, FH1 identifies essentially no women at high or very high risk.

The distributions of heritable risks (thin bars) are unaffected by the information provided to BOADICEA. These risks depend only on PV, PRS, and the unknown portion of the polygene. The only variation is at high risk, since only the threshold for defining high risk is varying down the rows, with results corresponding to those in Fig. 2 above. About 60% of women are at very low heritable risk (≤0.5), while over 70% are either very low or low risk (<0.8). The proportion at high heritable risk declines as the high-risk threshold increases. (Those at “very high” risk do not vary as this group is a subset of the high-risk group.) In all cases, women *assessed* as high or low risk comprise only a fraction of the corresponding *heritable* risk percentages. For example, while essentially no women are *assessed* as high risk in the absence of the PV and PRS information when Hi-RT ≥ 2.5, the percentages of women with *heritable* high risk ranges from 11.5% when Hi-RT = 2.0 to 8.8% at 2.5 to a bit over 5.7% when it is 3.5.

**Fig. 5:**
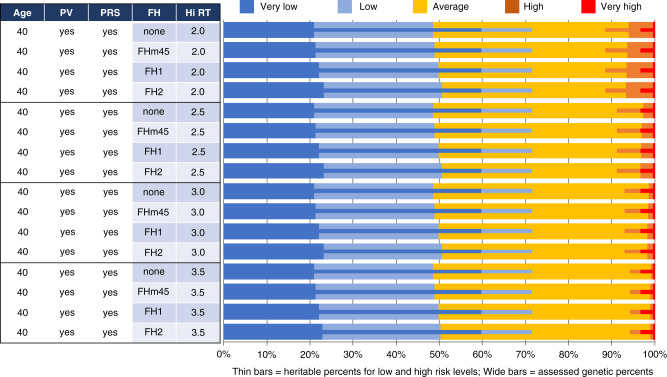
Univariate assessed and heritable breast cancer risk distributions by type of family history information provided to BOADICEA and by high-risk threshold (Hi-RT, the multiple of the age-specific absolute risk of breast cancer incidence). In all cases, the risk assessment is for women aged 40 and with both pathogenic variant (PV) and polygenic risk score (PRS) proband information provided to BOADICEA.

Figure 5 focuses on the kind of family history information provided to BOADICEA for risk assessment: (1) no FH information at all (“none”), (2) whether or not the woman’s mother had BC by age 45 (FHm45), (3) information for all first-degree relatives (FH1), and (4) information for all first- and second-degree relatives (FH2). In all scenarios, women have their risk assessed at age 40, and both PV and PRS information are considered at risk assessment.

At all four high-risk thresholds, the proportion of women identified as high risk increases as more detailed FH information is provided, though these increases are all relatively small. Further, providing more information on FH *increases* the proportions of women assessed as low and very low risk, since knowing of an *absence* of FH reduces the assessed risk.

**Fig. 6:**
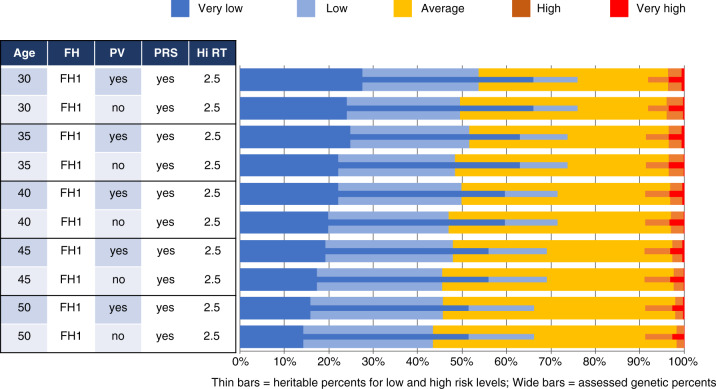
Univariate assessed and heritable breast cancer risk distributions by age at risk assessment and whether or not pathogenic (PV) information is provided to BOADICEA. In all cases, the risk assessment is for women with both polygenic risk score (PRS) and first-degree family history (FH1) information provided to BOADICEA. The high-risk threshold (HI-RT) in all cases is 2.5 (the multiple of the age-specific absolute risk of breast cancer incidence).

Figure 6 shows selected GMM results for varying ages at risk assessment, focusing on whether or not PV information is provided to BOADICEA, in addition to PRS and FH1, and Hi-RT = 2.5. The proportions of women defined as being at high *heritable* risk (rightmost thin bars) show almost no variation from age 30 to age 50. In contrast, the percentage *assessed* as high-risk declines with age, whether or not PV information is provided to BOADICEA. The assessed proportions at very low and low risk decline noticeably with increasing age at assessment, as do the proportions with very low and low heritable risk. However, even at age 50, the usual starting age for organized BC screening, about 15% of women have very low assessed genetic BC risks while over 40% would have assessed risks less than 0.8 times their age-specific population average. At all ages, including PVs in the risk assessment increases the proportions of women assessed as very low or low risk.

## DISCUSSION

There is growing interest in using women’s genetic risk information to personalize broad-based BC screening programs to their predicted risk, e.g., using the BOADICEA risk prediction tool. We have first developed and applied GMM to estimate the distributions of BC risk predictions for a plausibly realistic simulated population sample of Canadian women. We have then used GMM simulations to compare the impacts of using not only FH and PVs, but also the most recent PRS [1] for population-level risk assessments.

PVs, especially in *BRCA1* and *BRCA2*, have long been the focus for identifying women at the highest risk, along with FH. However, our analysis shows that at the population rather than individual level, PRS appears far more important for purposes of risk stratification for BC screening. Further, and contrary to some current thinking [12], information on FH appears of limited value as a risk stratifying tool at a population level, especially if this information is only dichotomous, such as whether or not a woman has a first-degree relative who has already had BC. More extensive FH information remains of limited importance; information on second-degree relatives is even less incrementally informative, while more expensive and difficult to collect accurately. FH is more informative in older than younger woman, since BC incidence, including in individuals at high risk and their relatives, increases with age.

This analysis has a number of limitations. BOADICEA [7] includes a range of other risk factors not considered, such as breast density, parity, and ages at menopause and menarche. The sensitivity of the results to the uncertainty in estimated prevalences for the PVs has not been explored. However, estimates from different studies are broadly similar, at least in European populations [13, 14]. The effect sizes for the PRS may also vary somewhat among different ethnicities [15]. While GMM is using recent Canadian demographic transition data, it generates a steady-state population distribution, not one reflecting historical and projected trends. Still, the results are likely to be qualitatively similar in a wide range of populations.

Randomized controlled screening studies that incorporate the PRS are ongoing [2], [16] and several recent simulation studies suggest considerable promise for enhanced breast cancer screening for women assessed as high risk, though without full consideration of the joint impacts of the three kinds of genetic information included in this study [4, 6]. The PERSPECTIVE I&I pilot study, currently underway [2, 17], will offer important new evidence on the practicalities of population-level risk assessment. As a result, with significant improvements in risk prediction, especially using a PRS and the BOADICEA risk prediction tool [7], the option of shifting to risk stratification for BC screening offers the prospect of improving the balance of benefits and harms [3–6, 18].

Further, identifying women at low risk offers the opportunity to avoid an important part of the costs and adverse effects of BC screening (e.g., false positives, overdiagnosis) by reducing their screening intensity. However, as highlighted in this study, only by including PRS in the risk assessment would this be possible.

Finally, the analysis presented pertains only to women and their risks for BC. Still, the incidence of both male and female BC, as well as ovarian, prostate and pancreatic cancer, is simulated for everyone in the population because the shared genetics of the proband’s family members affect her BC risk. This part of the simulation uses the same algorithms as BOADICEA.

### Conclusion

This analysis suggests that where there are tradeoffs, population-wide programs for BC screening that seek to stratify women by their genetic risk should focus first on PRS, not on more highly penetrant but rarer variants, nor family history. However, firmer conclusions must await embedding these GMM results in a fuller cost-effectiveness evaluation that includes BC screening, treatment, progression, mortality, and other harms and benefits (e.g., as in the Cancer Intervention and Surveillance Modeling Network (CISNET) models [4].

## Data Availability

The source code for GMM is accessible on https://ompp.sourceforge.io/wiki/index.php/Main_Page. GMM can be run provided it is for research purposes, and permission has been granted by the authors. The BOADICEA v.5 code can be obtained for research purposes from the authors.
